# SPHK1 drives pyroptosis via ROS-dependent activation of the NLRP3 inflammasome in non-infectious uveitis

**DOI:** 10.3389/fimmu.2026.1764448

**Published:** 2026-09-29

**Authors:** Lili Hu, Shaoxin Pan, Jing Wang, Suyin Mei, Haoning Pan, Rui Chang

**Affiliations:** 1 Ophthalmology Medical Center, Chongqing Key Laboratory for the Prevention and Treatment of Major Blinding Eye Diseases, The First Affiliated Hospital of Chongqing Medical University, Chongqing Branch (Municipality Division) of National Clinical Research Centre for Ocular Diseases, Chongqing, China; 2 Ophthalmology Medical Center, The Second Affiliated Hospital of Wannan Medical College, Wuhu, Anhui, China; 3 Ophthalmology Medical Center, Beijing Shijitan Hospital Affiliated to Capital Medical University, Beijing, China; 4 Wannan Medical College, Wuhu, Anhui, China

**Keywords:** Behçet’s disease, non-infectious uveitis, oxidative stress, pyroptosis, sarcoidosis, spondyloarthropathy

## Abstract

**Introduction:**

Non-infectious uveitis associated with systemic diseases is a sight-threatening inflammatory disorder often associated with dysregulated immune responses and oxidative stress. Although redox imbalance is thought to contribute to its pathogenesis, the precise molecular mechanisms remain poorly defined.

**Methods:**

We performed transcriptomic analysis of human non-infectious uveitis datasets (GSE17114 and GSE18781) encompassing Behçet's uveitis (BU), sarcoidosis-associated uveitis, and spondyloarthropathy (SpA)-associated uveitis, combined with experimental validation to elucidate oxidative stress-associated mechanisms.

**Results:**

We identified 1,821 differentially expressed genes (DEGs) enriched in immune cell migration, T and B cell signaling, and T cell activation pathways. Among the oxidative stress-associated DEGs, six genes were validated in patients with BU: SPHK1, MMP8, and ARG1 were upregulated, while CCR7, PDGFD, and DDIT4 were downregulated. Knockdown of SPHK1 reduced ROS production, lipid peroxidation, and NLRP3 inflammasome-mediated pyroptosis, decreasing IL-1β and IL-18 secretion; SPHK1 overexpression promoted pyroptosis, reversed by the ROS inhibitor N-acetylcysteine. Molecular docking revealed strong binding affinity between SPHK1 and dexamethasone. All six oxidative stress-related DEGs demonstrated strong diagnostic potential. Immune cell abundance profiling revealed a significant decrease in activated CD4+ T cells, memory B cells, and NK cells in peripheral blood.

**Discussion:**

Collectively, our findings highlight SPHK1 as a key mediator of oxidative stress-induced pyroptosis in non-infectious uveitis and propose the SPHK1–ROS–pyroptosis axis as a potential therapeutic target.

## Introduction

Uveitis encompasses a diverse group of intraocular immune-mediated inflammatory disorders that primarily affect the uveal tract, retina, and vitreous body, with more than 100 clinically distinct subtypes (1). The condition most frequently affects young and middle-aged adults, often pursuing a chronic or relapsing course that severely impact quality of life (2). Notably, nearly one-third of all uveitis cases are associated with systemic autoimmune diseases —a finding corroborated by our recent large-scale cohort study of 15, 373 patients, which identified Behçet’s disease (BD), Vogt-Koyanagi-Harada (VKH) syndrome, sarcoidosis, and spondyloarthropathies as the most common comorbidities (3). Uveitis remains a leading cause of irreversible vision loss, responsible for 10% to 15% of blindness in developed countries and up to 25% in regions with limited resources (4, 5). Despite this substantial disease burden, current management remains suboptimal. First-line therapies such as glucocorticoids and immunosuppressive agents are constrained by significant systemic toxicity, the emergence of drug resistance, and considerable inter-patient variability in therapeutic response (6, 7). These challenges underscore the urgent need to clarify the molecular drivers of uveitis and to develop more effective diagnostic and therapeutic strategies. The present study focuses specifically on non-infectious uveitis—particularly the immune-mediated subtypes associated with systemic diseases, including Behçet’s disease, sarcoidosis, and spondyloarthropathy. Infectious etiologies, such as viral (e.g., cytomegalovirus, herpes simplex), bacteria (e.g., tuberculosis, syphilis), fungal, and parasitic (e.g., toxoplasmosis) causes, are explicitly excluded from this investigation. This distinction is critical: infectious and non-infectious uveitis differ fundamentally in their pathogenesis, clinical presentation, and therapeutic approaches, and conflating them in molecular studies can obscure disease-specific signatures (8, 9). By restricting our analysis to well-characterized non-infectious subtypes, we aimed to minimize etiological heterogeneity and enhance the translational relevance of our findings. Given that Behçet’s disease, sarcoidosis, and spondyloarthropathy differ substantially in their anatomical distribution, immunopathogenic mechanisms, and clinical severity, each disease group was analyzed separately rather than pooled into a single uveitis group, so that shared molecular signatures were identified empirically across disease groups rather than assumed *a priori* to be identical.

Disruption of redox homeostasis, characterized by persistent oxidative stress, is a fundamental driver of immune-inflammatory pathology (10). For example, in systemic lupus erythematosus (SLE), rheumatoid arthritis (RA), and multiple sclerosis, decreased activities of antioxidant enzymes, including superoxide dismutase and glutathione peroxidase, together with increased reactive oxygen species (ROS) and elevated lipid peroxidation products such as malondialdehyde, have all been shown to correlate with disease activity (11–13). Furthermore, depletion of thioredoxin and glutathione disrupts immune balance, promoting pathogenic T helper 17 (Th17) polarization in SLE and RA (14, 15). The release of tumor necrosis factor (TNF) and interleukin-1 (IL-1) by activated macrophages and T cells triggers the activation of ROS and NF-κB signaling, initiating inflammatory gene expression and perpetuating a vicious cycle of oxidative tissue damage, particularly in RA (15). Meta-analyses of transcriptomic data have demonstrated a conserved upregulation of genes related to oxidative stress and inflammation across various autoimmune diseases. *In vivo* studies further support the therapeutic value of antioxidants in models of RA and other inflammatory disorders (16). Our previous investigations have directly linked redox imbalance to uveitis pathogenesis. We showed that patients with VKH and Behçet’s disease have increased ROS production and activation of the NLRP3 inflammasome, along with higher levels of pro-inflammatory cytokines such as IL-1β, TNF-α, and IL-6 in monocyte-derived macrophages (17, 18). Nevertheless, the molecular mediators that connect oxidative stress with immune dysfunction in uveitis remain incompletely defined. In particular, both the upstream regulatory mechanisms that couple redox imbalance to immune cell activation and the downstream effector pathways through which oxidative stress drives ocular tissue damage have not been systematically characterized, representing a significant barrier to the development of precise, targeted treatments for vision-threatening inflammation.

To address this gap, we performed an integrated analysis combining transcriptomic profiling of autoimmune uveitis datasets (GSE17114 and GSE18781) with experimental validation (19, 20). Our study identified six pivotal oxidative stress-related genes, including MMP8, ARG1, SPHK1, DDIT4, CCR7, and PDGFD, as potential mediators of disease. Among these, SPHK1 — which encodes sphingosine kinase 1, the rate-limiting enzyme that phosphorylates sphingosine to sphingosine-1-phosphate (S1P) (21) — is of particular interest, given its established roles in inflammatory signaling, oxidative stress responses, and immune cell trafficking across autoimmune and inflammatory diseases (22–24). Functional experiments demonstrated that SPHK1 promotes the production of ROS, which subsequently activates the NLRP3 inflammasome and induces caspase-1-dependent pyroptosis, a form of cell death that intensifies inflammatory responses in uveitis. These results indicate that SPHK1 and its related signaling pathways may serve as promising molecular targets for therapeutic intervention, offering new opportunities to alleviate intraocular inflammation and enhance clinical outcomes in this challenging group of diseases.

## Methods

### Data collection

Transcriptomic data were retrieved from the Gene Expression Omnibus (GEO, https://www.ncbi.nlm.nih.gov/geo/). Dataset GSE17114, generated on the GPL570 platform (Affymetrix Human Genome U133 Plus 2.0 Array), included peripheral blood mononuclear cell (PBMC) samples from four patients with active Behçet’s uveitis (BU) and fourteen healthy controls. Eleven Behçet’s disease samples without ocular involvement, included in the same dataset, were excluded to isolate uveitis-specific transcriptional signatures (19). Patients were considered immunosuppressed (IS) if they were receiving azathioprine, cyclosporine, deflazacort, or thalidomide at the time of blood collection; three of the four BU samples were IS and one was non-IS. Dataset GSE18781, also profiled on the GPL570 platform, comprised whole-blood RNA samples from twenty-one patients with non-infectious uveitis, including seven cases of sarcoidosis-associated uveitis and fourteen cases of spondyloarthropathy (SpA)-associated uveitis, alongside twenty-five healthy controls. Nine non-uveitis disease samples from this dataset were excluded from our analyses (20). In GSE18781, SpA was diagnosed using a likelihood-ratio score as described by Rudwaleit and colleagues; other autoimmune diseases were excluded (ulcerative colitis and psoriasis were permitted as recognized SpA co-morbidities); and systemic immunomodulatory therapy was not permitted, with only one patient having previously received a TNF inhibitor (etanercept), which was discontinued two months before blood collection. A curated list of oxidative stress-related genes was retrieved from the Molecular Signatures Database (MSigDB; http://www.gsea-msigdb.org) for subsequent analyses (**Supplementary Table S1**).

### Differentially expressed genes analysis

Differentially expressed genes (DEGs) between the uveitis and control groups were identified using the R package limma (version 3.50.0). Each disease group was compared separately against healthy controls—BU versus controls in GSE17114, and sarcoidosis/SpA-associated uveitis versus controls in GSE18781—rather than pooling all uveitis samples into a single group. Genes with an absolute log2 fold change greater than 0.5 and a p-value below 0.05 were considered significantly differentially expressed. Oxidative stress-related DEGs (OSDEGs) were determined by intersecting the identified DEGs with the MSigDB-derived oxidative stress gene set, ensuring a focus on genes relevant to redox biology.

### Functional enrichment analysis

Functional enrichment analysis was conducted using Gene Ontology (GO) and Kyoto Encyclopedia of Genes and Genomes (KEGG) pathways with the clusterProfiler R package (version 4.2.2). GO enrichment was evaluated across three domains: biological process (BP), molecular function (MF), and cellular component (CC). GO and KEGG terms with Benjamini–Hochberg-adjusted p-values less than 0.05 were deemed statistically significant, ensuring stringent control for multiple testing.

### Protein–protein interaction network analysis

To investigate molecular interactions among the OSDEGs, protein–protein interaction (PPI) networks were constructed using the GeneMANIA platform (http://genemania.org). Co-expressed partner genes were selected from GeneMANIA’s multi-network integration (co-expression, physical interaction, shared protein domains, co-localization, pathway, and genetic interaction networks) ranked by composite score in descending order; the top 20 genes by score were retained as co-expressed partners. Subsequent enrichment analysis of these co-expressed genes was conducted using clusterProfiler, enabling the identification of critical signaling pathways and molecular complexes implicated in the pathogenesis of uveitis.

### Single-gene gene set enrichment analysis

Single-gene gene set enrichment analysis (GSEA) was conducted for selected OSDEGs using clusterProfiler and the c2.cp.kegg.v7.2.symbols gene set from MSigDB. The analysis was performed with 1, 000 permutations, considering gene sets ranging in size from 10 to 500 genes, and significance was defined by an adjusted p-value below 0.05. This approach allowed for the precise assessment of pathway involvement for individual key genes.

### Diagnostic value assessment

The diagnostic potential of the candidate genes was assessed by generating receiver operating characteristic (ROC) curves using the pROC R package (version 1.18.0). The area under the curve (AUC) was calculated to quantify diagnostic accuracy, allowing for direct comparison of gene performance across different cohorts.

### Immune cell abundance profiling

Relative immune cell-type abundances in peripheral blood transcriptomes were estimated using single-sample gene set enrichment analysis (ssGSEA), based on 28 predefined immune cell gene sets obtained from the TISIDB database (https://cis.hku.hk/TISIDB/). The results were visualized using the ggplot2 package (version 3.3.6). It should be noted that this analysis estimates the relative proportions of immune cell types from gene expression signatures and does not directly measure immune cell migration into ocular tissues; accordingly, the term “immune cell abundance profiling” is used throughout to distinguish this computational deconvolution approach from direct measurement of immune cell infiltration. This analysis provided a detailed profile of immune cell-type composition in uveitis and control peripheral blood samples, supporting the identification of disease-specific immune signatures.

### Transcription factor regulatory network

Transcription factor (TF)–mRNA interactions were obtained from the hTFtarget database. The resulting regulatory networks were visualized using Cytoscape software (version 3.9.1), enabling the identification of potential upstream regulators of OSDEG expression.

### Competing endogenous RNA network

To construct ceRNA networks, potential miRNA–mRNA–lncRNA interactions were predicted using miRTarBase, StarBase 2.0, and miRDB. This approach facilitated the exploration of post-transcriptional regulatory mechanisms influencing OSDEG activity.

### RNA-binding protein interaction network

RBP–mRNA interactions were identified using StarBase CLIP-seq data, applying stringent criteria (p < 0.05 and cluster number ≥ 5) to ensure reliability. This network analysis enabled the discovery of additional layers of post-transcriptional regulation.

### Collection of biospecimens and qRT-PCR analysis

Peripheral blood samples were obtained from patients with Behçet’s uveitis (BU) and matched healthy controls at the Ophthalmology Medical Center, The First Affiliated Hospital of Chongqing Medical University. Inclusion criteria were: (i) a confirmed diagnosis of BU according to the 1990 International Study Group criteria (25); (ii) active ocular inflammation at the time of sampling; and (iii) blood drawn before the initiation of any new therapeutic intervention. Exclusion criteria were: (i) current or recent systemic immunomodulatory or immunosuppressive therapy; (ii) concurrent ocular disease, including infectious uveitis and other non-infectious uveitis entities; (iii) other systemic autoimmune or inflammatory diseases; (iv) malignancy; and (v) active infection. Healthy controls were matched by age, sex, and ethnicity and were free of ocular and systemic inflammatory disease. All procedures adhered to the Declaration of Helsinki, with informed consent obtained from all subjects. Peripheral blood mononuclear cells (PBMCs) were isolated using Ficoll density gradient centrifugation. Total RNA was extracted using TRIzol reagent, reverse-transcribed into cDNA, and quantified via SYBR Green-based qRT-PCR. Gene expression levels were normalized to GAPDH and calculated using the 2^−ΔΔCt method.

### Cell culture and functional experiments

Bone marrow-derived macrophages (BMDMs) were used as they are the principal innate immune effector cells of the uveal tract (26), the primary cell type in which the NLRP3 inflammasome/pyroptosis machinery operates, and a cell type in which SPHK1 is known to regulate NLRP3 inflammasome activation; they were stimulated with LPS, the canonical priming signal that licenses NLRP3 inflammasome activation, to recapitulate the ROS–NLRP3–pyroptosis axis identified in our transcriptomic screen. BMDMs were purchased from a commercial supplier (Wuhan Pulosai Biotechnology Co., Ltd., which isolated them from C57BL/6 mice) and cultured in DMEM supplemented with 10% fetal bovine serum and 20 ng/mL macrophage colony-stimulating factor (M-CSF) for seven days. To establish an *in vitro* model of uveitis-relevant inflammation, BMDMs were stimulated with lipopolysaccharide (LPS, 100 ng/mL) for eight hours. This system serves as the *in vitro* correlate of the endotoxin-induced uveitis (EIU) model and has been widely validated for studying NLRP3 inflammasome-mediated pyroptosis in macrophages (27). SPHK1 knockdown was performed using siRNA transfection with Lipofectamine 3000, while overexpression was induced by transfecting a pcDNA3.1-SPHK1 plasmid. For rescue experiments, cells were pretreated with the ROS scavenger N-acetylcysteine (NAC, 5 mM) two hours prior to LPS stimulation. Intracellular ROS levels were measured using DCFH-DA fluorescence, lipid peroxidation was assessed by quantifying malondialdehyde (MDA), and cytokine levels (IL-1β and IL-18) were determined by ELISA. Protein expression of NLRP3, ASC, cleaved caspase-1, and GSDMD was analyzed via Western blotting.

### Molecular docking

Molecular docking was performed using the CB-Dock2 web server (http://cao.labshare.cn/cb-dock2/). The three-dimensional (3D) structure of the protein target SPHK1 was obtained from the Protein Data Bank (PDB ID: 4V24). The 3D structure of the small molecule ligand, Dexamethasone (CID: 5743), was retrieved from the PubChem database. Blind docking was carried out using the AutoDock Vina program embedded within the CB-Dock2 platform. The binding affinity is reported as a Vina Score (kcal/mol), where a score more negative than -7.0 kcal/mol indicates strong binding, a score between -7.0 and -4.0 kcal/mol suggests moderate binding, and a score greater than -4.0 kcal/mol signifies weak or no significant binding.

### Statistical analysis

All transcriptomic analyses were conducted in R (version 4.1.2). Differential gene expressions were assessed using the limma package, while Spearman correlation was used for association analyses. Group comparisons were performed using the Wilcoxon rank-sum test or Kruskal-Wallis test, as appropriate. Experimental validation data, obtained from at least three independent biological replicates, were expressed as mean ± standard deviation (SD) and analyzed using GraphPad Prism 9.0. The Shapiro-Wilk test was used to assess data normality. For comparisons between two groups, unpaired t-tests were applied to parametric data, and Mann–Whitney U tests to nonparametric data. For multiple group comparisons, one-way ANOVA followed by Tukey’s *post hoc* test or the Kruskal–Wallis test with Dunn’s multiple comparisons was used. A two-sided p-value < 0.05 was considered statistically significant.

## Results

### Identification and functional enrichment analysis of DEGs

The overall workflow of this study is illustrated in **Figure 1**. Each disease group was analysed separately against healthy controls—BU in GSE17114 and sarcoidosis/SpA-associated uveitis in GSE18781—rather than pooling all uveitis samples into a single group. Analysis of the GEO datasets identified 660 significant DEGs between BU samples and healthy controls (P < 0.05, |log2FC| > 0.5), with 337 genes upregulated and 323 downregulated (**Supplementary Table S2**). A volcano plot depicts the global distribution of DEGs (**Figure 2A**), while a heatmap displays the top five most upregulated and downregulated genes in BU samples (**Figure 2B**). In the sarcoidosis and SpA uveitis group, 1, 161 DEGs were identified, including 160 upregulated and 1, 001 downregulated genes (**Supplementary Table S3**), as shown in **Figures 2C, D**. Enrichment analysis of BU-related DEGs revealed significant involvement in biological processes such as myeloid leukocyte activation, cell killing, and cytokine production. Cellular component analysis highlighted enrichment in the specific granule lumen and tertiary granules, while molecular function analysis pointed to proteoglycan binding, MHC class I receptor activity, immune receptor activity, and signaling pathways involving T and B cell receptors and chemokines (**Figures 2E–G**). In sarcoidosis/SpA-associated uveitis, DEGs were primarily enriched in T cell activation and differentiation processes, components like the immunological synapse and preribosome, and KEGG pathways including T cell receptor signaling, hematopoietic cell lineage, Th1/Th2/Th17 cell differentiation, and PD-1/PD-L1 checkpoint signaling (**Figures 2H–J**).

**Figure 1:**
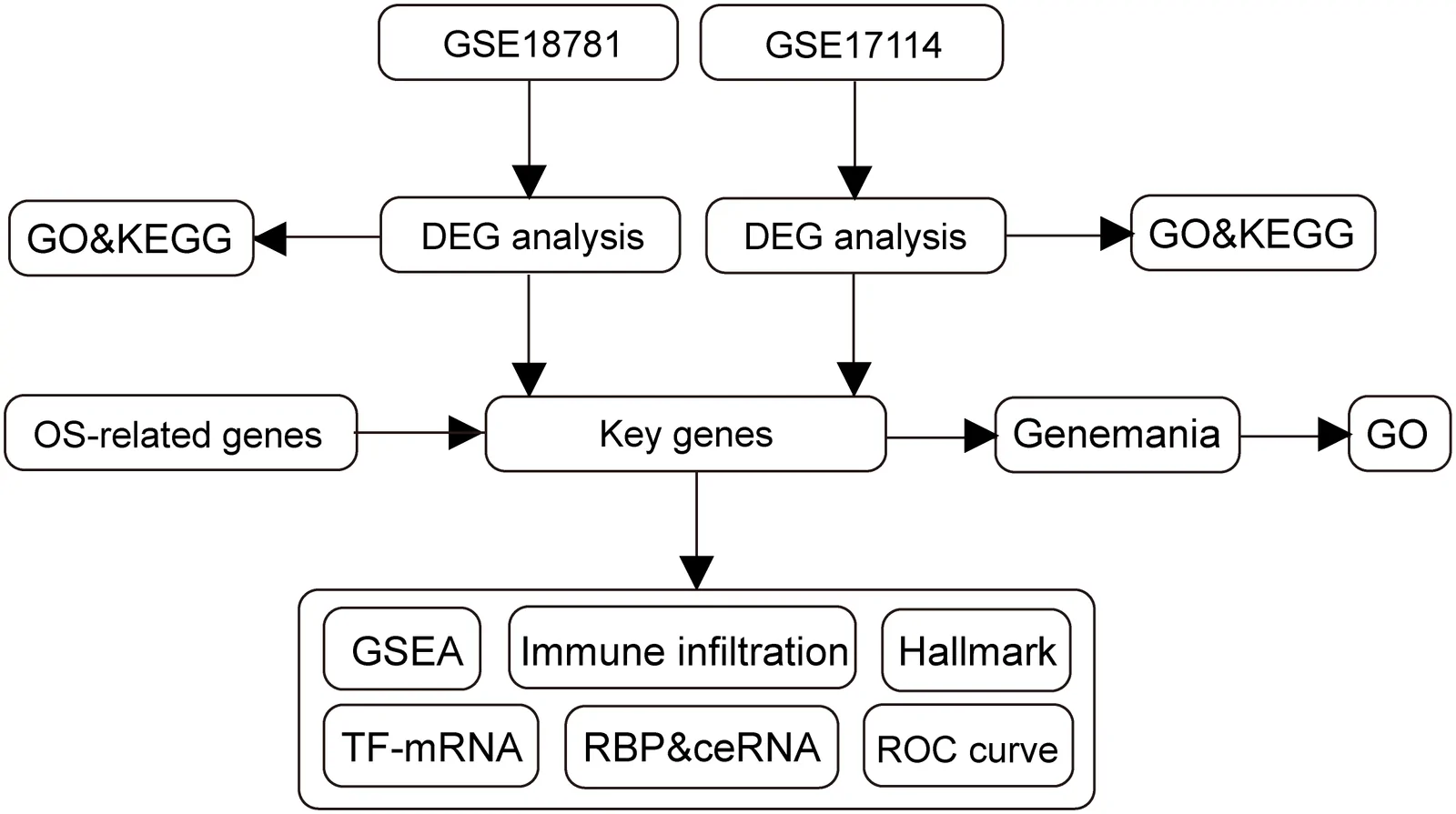
Study design flowchart. An overview of the study design and workflow. DEGs, Differentially Expressed Genes; OS-related genes, oxidative stress-related genes; GO, Gene Ontology; KEGG, Kyoto Encyclopedia of Genes and Genomes; GSEA, Gene Set Enrichment Analysis; TF, transcription factors; RBP, RNA-binding proteins; ROC, Receiver Operating Characteristic.

**Figure 2:**
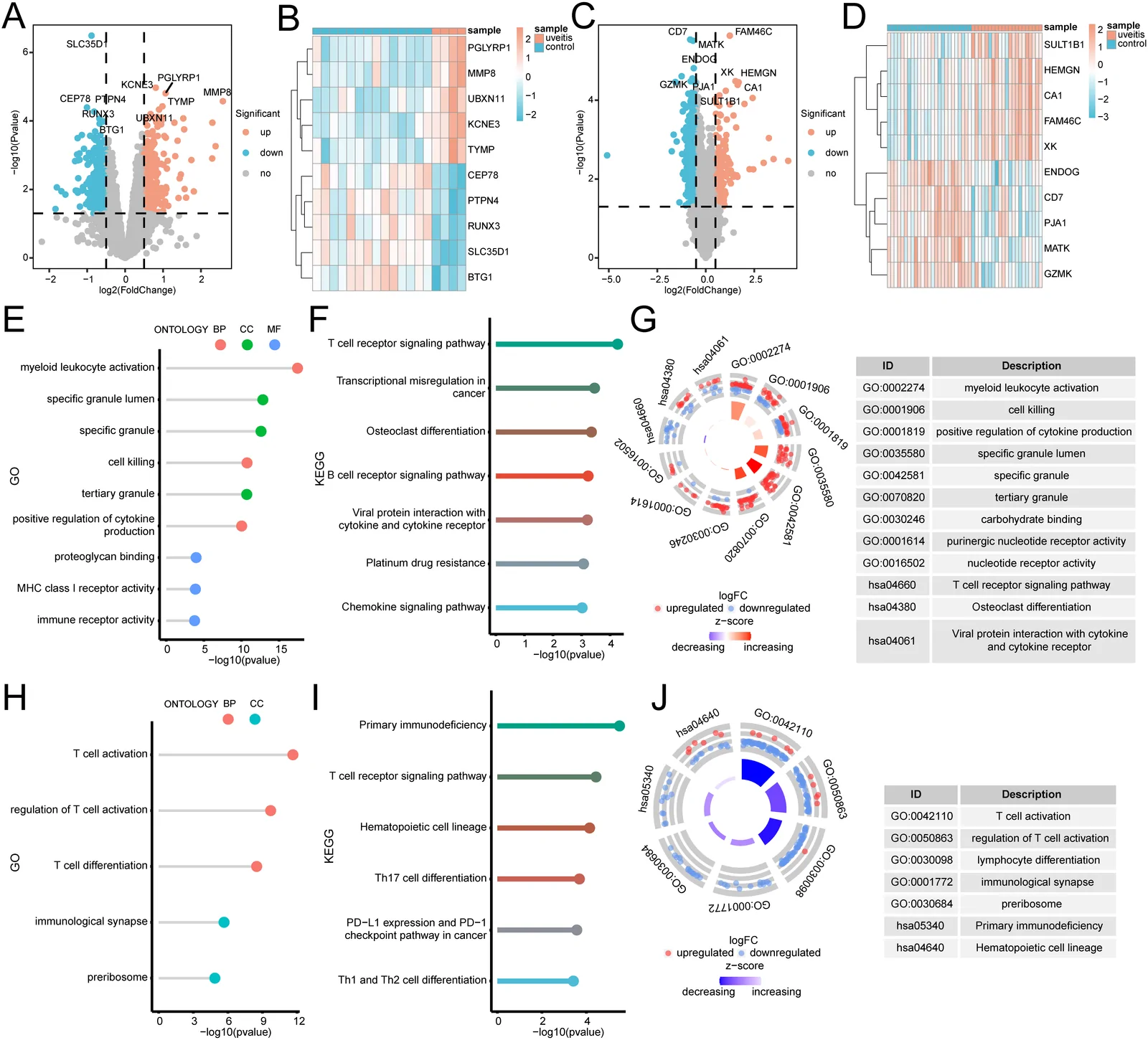
Enrichment analysis of differentially expressed genes. **(A, C)** Volcano plots display DEGs in Behçet’s disease (BD) uveitis and sarcoidosis/spondyloarthropathy (SpA) uveitis versus controls, with red and blue dots indicating upregulated and downregulated genes, respectively. **(B, D)** Heatmaps show the top 10 DEGs in BD and sarcoidosis/SpA uveitis. **(E, F)** GO and KEGG enrichment analyses for BD uveitis DEGs are presented in circular layouts: outer scatterplots show gene logFC values (red: upregulated); inner bar charts display enrichment significance (color intensity reflects z-score). **(G)** Integrated visualization of GO and KEGG enrichment for BD uveitis. **(H-J)** Corresponding GO and KEGG enrichment analyses for sarcoidosis/SpA uveitis DEGs, following the same visualization scheme.

### Identification and functional enrichment analysis of OSDEGs

A total of six OSDEGs, namely MMP8, DDIT4, CCR7, ARG1, PDGFD, and SPHK1, were identified (**Figure 3A**). Their expression patterns and correlations across different uveitis subtypes are presented in **Figures 3B–E**. PPI network analysis identified 26 genes in total—the six OSDEGs plus 20 co-expressed partner genes selected by GeneMANIA score ranking (**Supplementary Table S4**; **Figure 3F**). GO enrichment analysis of these co-expressed genes revealed significant involvement in biological processes including leukocyte migration, neutrophil chemotaxis, and RAGE receptor binding (**Figure 3G**; **Supplementary Table S5**).

**Figure 3:**
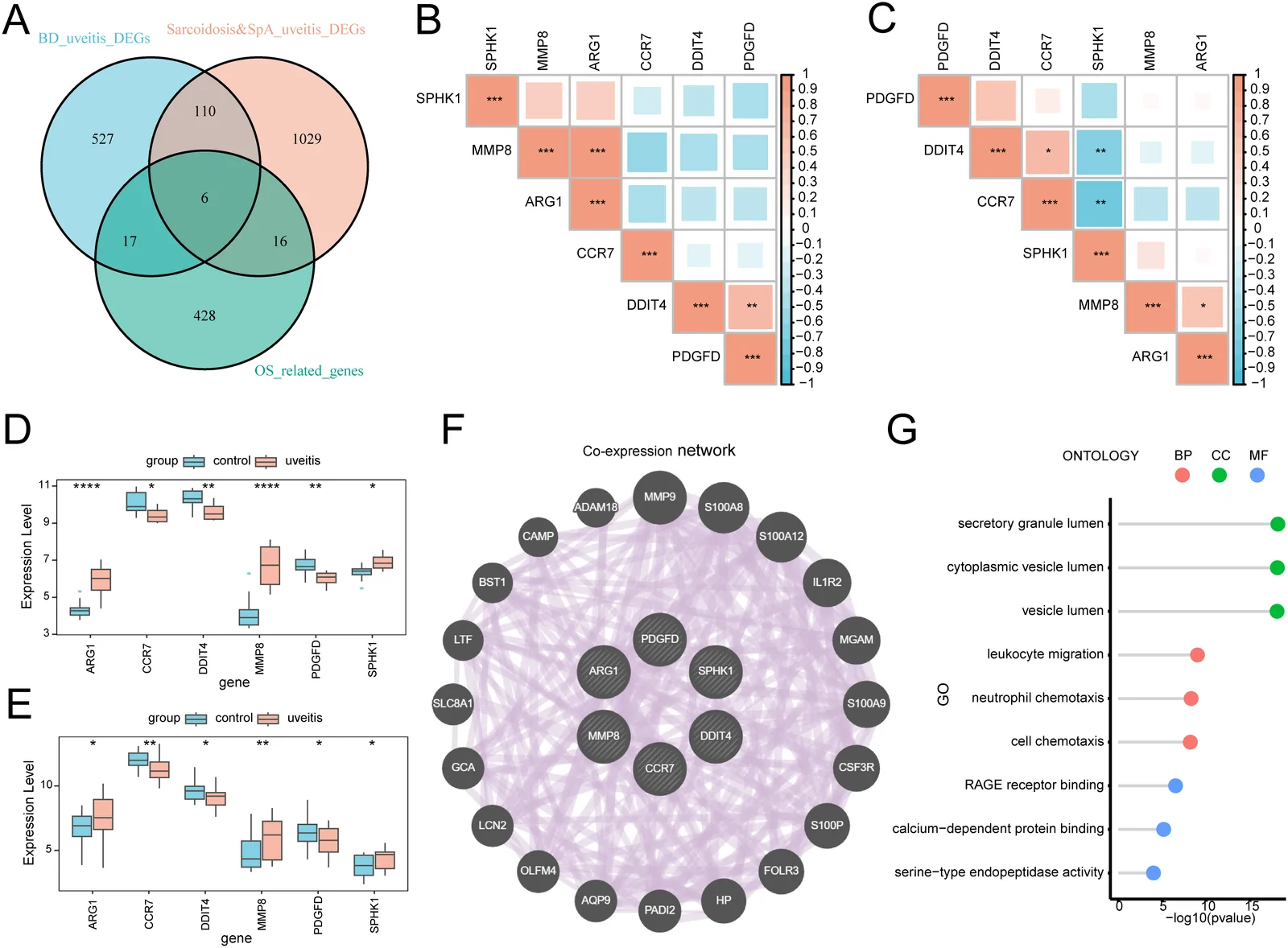
Screening and enrichment analysis of key genes. **(A)** Venn diagram of key genes. **(B, C)** Correlation analysis of key genes in the BD uveitis and Sarcoidosis uveitis cohorts. **(D, E)** Box plots showing expression levels of key genes in BD and Sarcoidosis uveitis samples. **(F)** Protein interaction network of co-expressed key genes. **(G)** GO enrichment analysis of co-expressed genes, represented as a lollipop chart. **Asterisks denote significance levels: *p < 0.05, **p < 0.01, ***p < 0.001, ****p < 0.0001.

### Single-gene gene set enrichment analysis

In the sarcoidosis and SpA cohort, single-gene GSEA showed that four OSDEGs were significantly enriched in specific biological pathways. Specifically, PDGFD-related genes were enriched in cell cycle regulation, Huntington’s disease, spliceosome function, and RNA degradation (**Figure 4A**). SPHK1-associated genes showed enrichment in Huntington’s disease, oxidative phosphorylation, spliceosome function, and RNA degradation (**Figure 4B**). CCR7-related genes were linked to Huntington’s disease, oxidative phosphorylation, T cell receptor signaling, and RNA degradation (**Figure 4C**). MMP8-related genes were enriched in the spliceosome, RNA degradation, primary immunodeficiency, DNA replication, and mismatch repair (**Figure 4D**). In Behçet’s disease–associated uveitis, genes correlated with ARG1 were enriched in pathways including porphyrin and chlorophyll metabolism, the citrate (TCA) cycle, glycerophospholipid metabolism, small cell lung cancer, and the chemokine signaling pathway (**Figure 4E**). Genes linked to PDGFD were enriched in graft versus host disease, Nod-like receptor signaling, ErbB signaling, and type I diabetes mellitus pathways (**Figure 4F**).

**Figure 4:**
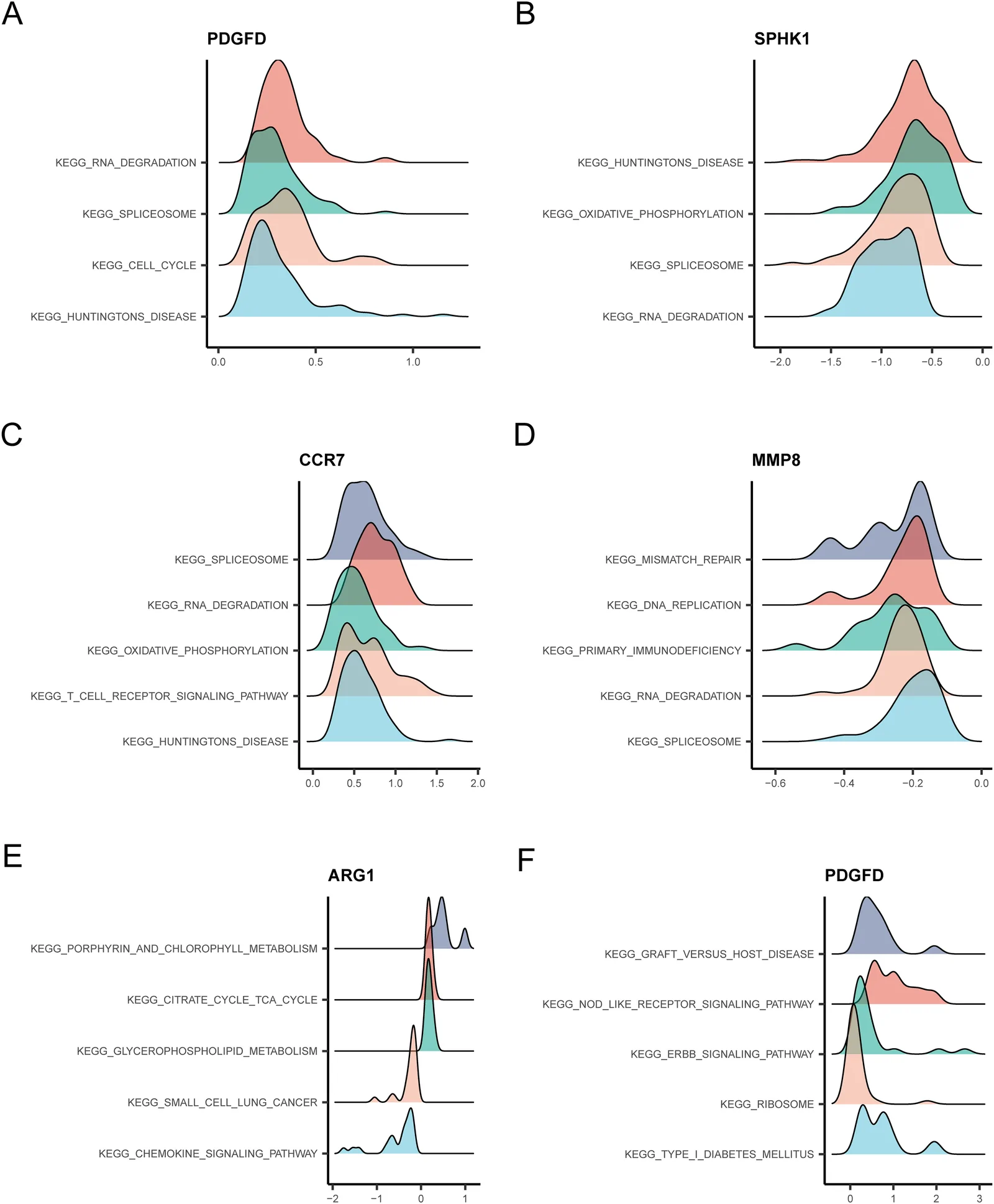
Single-gene gene set enrichment analysis of key genes. **(A)** GSEA for PDGFD, **(B)** SPHK1, **(C)** CCR7, and **(D)** MMP8 in the Sarcoidosis & SpA uveitis cohort. **(E)** ARG1 and **(F)** PDGFD GSEA in the BD uveitis cohort. GSEA, Gene Set Enrichment Analysis.

### Diagnostic evaluation of OSDEGs

The diagnostic performance of the six OSDEGs was assessed using ROC curve analysis in BU (**Figures 5A–F**) and sarcoidosis & SpA uveitis (**Figures 5G–L**). All six genes showed significant diagnostic value, with AUC values exceeding 0.6. Several of them achieved AUCs above 0.9, indicating excellent discriminatory power for detecting disease.

**Figure 5:**
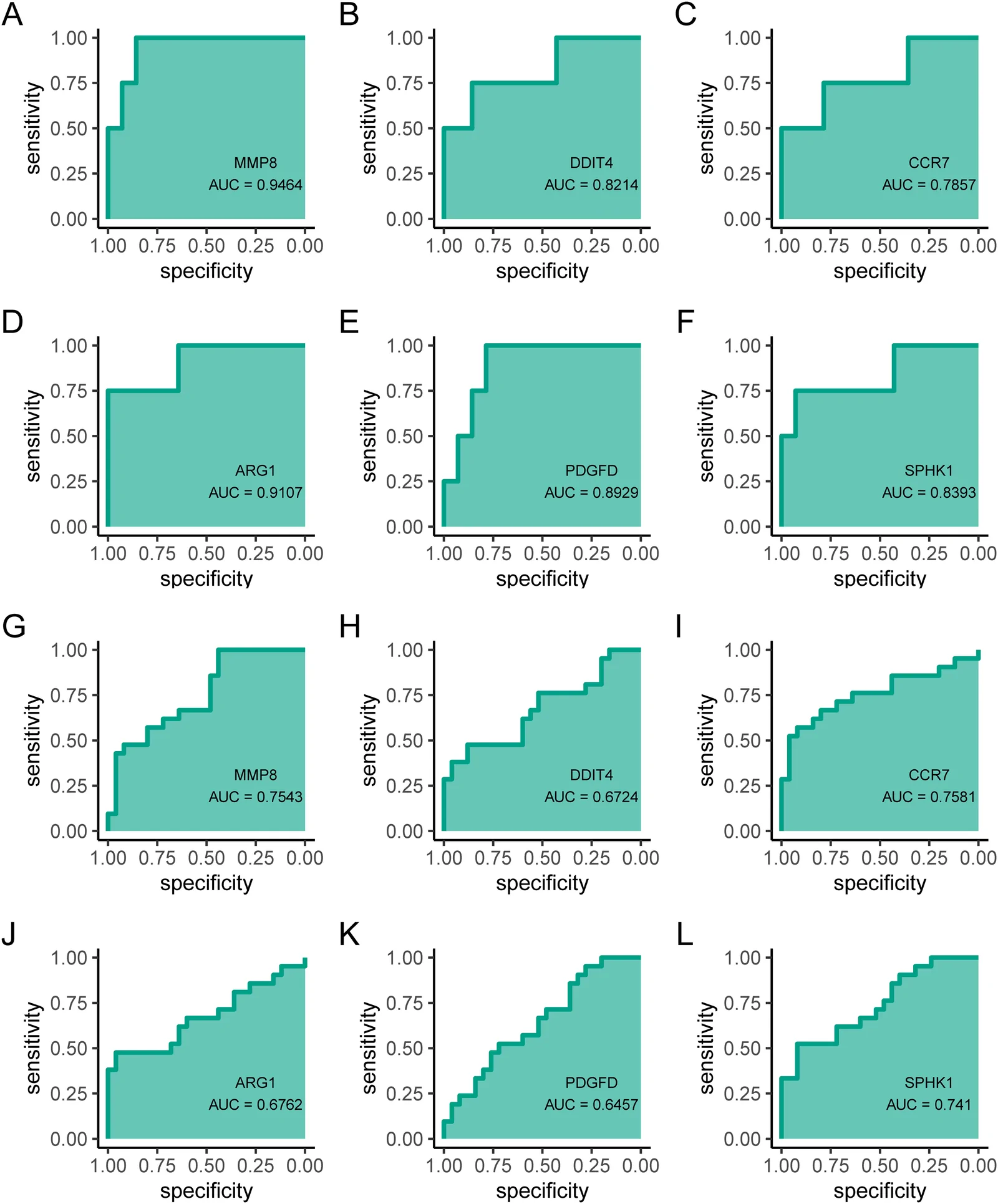
Diagnostic efficacy of key genes. ROC curves for key genes in BD uveitis cohort: **(A)** MMP8, **(B)** DDIT4, **(C)** CCR7, **(D)** ARG1, **(E)** PDGFD, and **(F)** SPHK1. ROC curves in Sarcoidosis & SpA uveitis cohort: **(G)** MMP8, **(H)** DDIT4, **(I)** CCR7, **(J)** ARG1, **(K)** PDGFD, and **(L)** SPHK1. ROC, Receiver Operating Characteristic.

### Hallmark pathway enrichment of DEGs

Gene set variation analysis (GSVA) was employed to compare the activity of 50 Hallmark signaling pathways between patients with BU and healthy controls (**Figure 6**), as well as between sarcoidosis & SpA uveitis patients and controls (**Figure 7**). In both cohorts, there was consistent upregulation of xenobiotic metabolism, ROS pathway, and complement activation, while the E2F Targets pathway was downregulated. OSDEGs exhibited significant correlations with these pathways, highlighting their involvement in oxidative stress-driven immune dysregulation and inflammation in uveitis.

**Figure 6:**
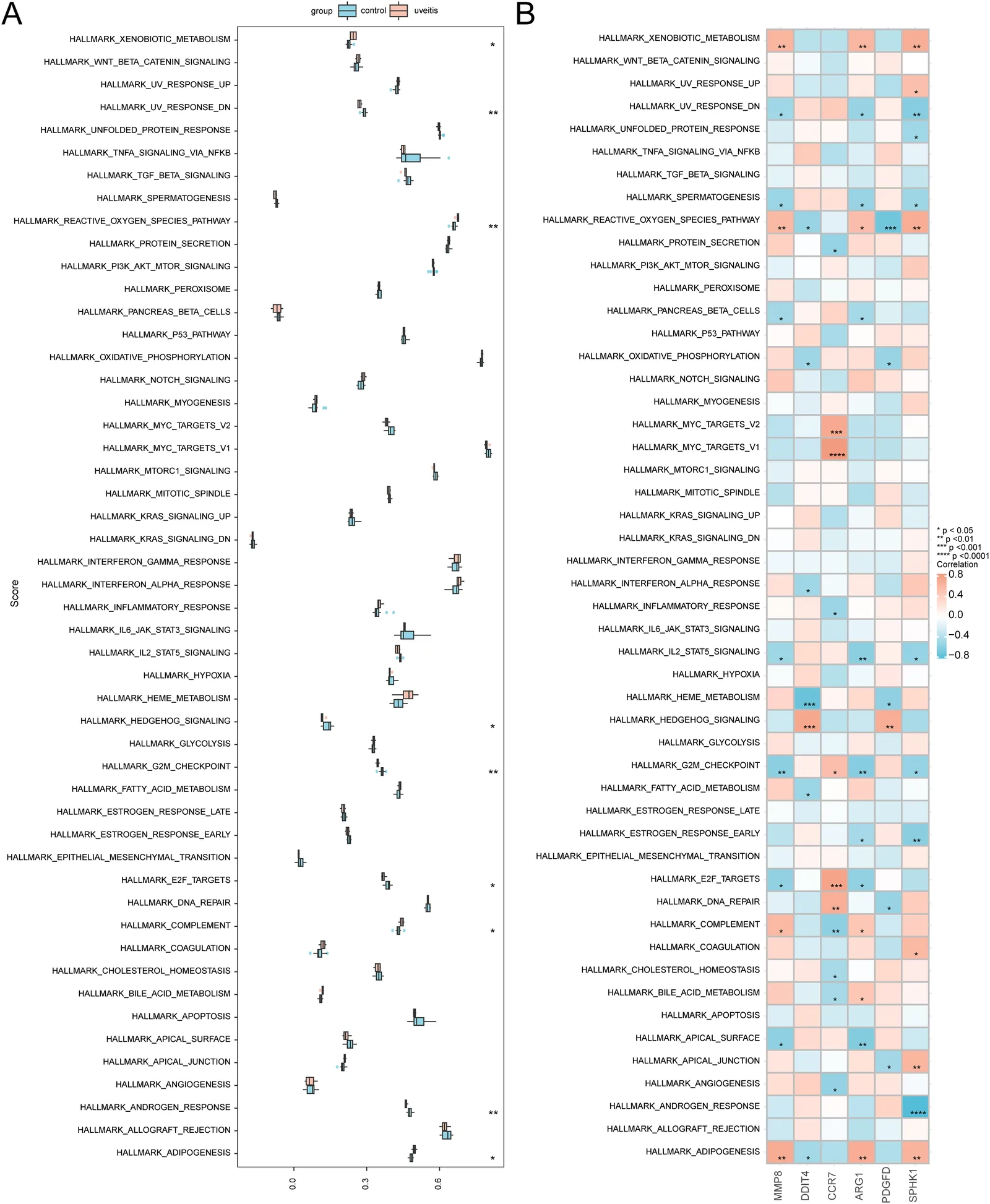
Correlation of BD uveitis key genes with 50 Hallmark signaling pathways. **(A)** Comparison of Hallmark pathway expression between BD uveitis and control groups. **(B)** Correlation of key genes with 50 Hallmark pathways. Asterisks denote significance levels: *p < 0.05, **p < 0.01, ***p < 0.001, ****p < 0.0001.

**Figure 7:**
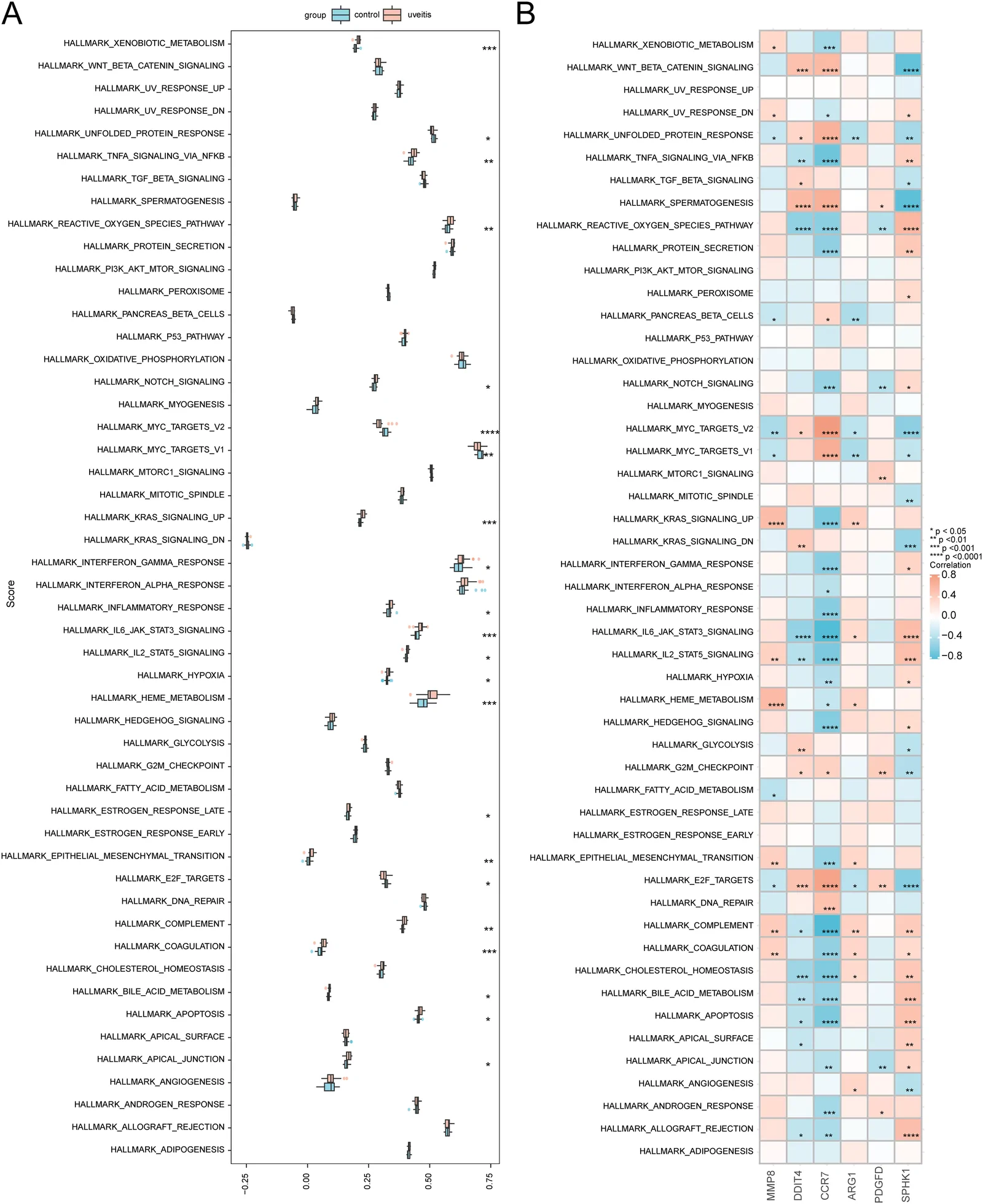
Correlation of sarcoidosis & SpA uveitis key genes with 50 Hallmark signaling pathways. **(A)** Comparison of Hallmark pathway expression between Sarcoidosis & SpA uveitis and control groups. **(B)** Correlation of key genes with 50 Hallmark pathways. Asterisks denote significance levels: *p < 0.05, **p < 0.01, ***p < 0.001, ****p < 0.0001.

### Immune cell abundance patterns and correlation analysis

To characterize the peripheral blood immune landscape in uveitis, we evaluated immune cell-type abundances in BU and sarcoidosis/SpA-associated uveitis samples using ssGSEA (**Supplementary Tables 6**, **S7**). Stacked bar plots confirmed intragroup consistency with no outlier samples detected (**Figures 8A**, **9A**). Comparative analysis revealed marked differences in peripheral blood immune cell composition between uveitis and control groups, with the relative abundances of activated CD4^+^ T cells, memory B cells, and NK cells consistently reduced across all three uveitis subtypes (**Figures 8C**, **9C**). Correlation analysis further demonstrated significant interrelationships among immune cell types within each disease group (**Figures 8B**, **9B**). To explore the functional relevance of oxidative stress-related DEGs (OSDEGs) to the immune landscape, we correlated their expression with immune cell abundances. Across all subtypes, ARG1 showed negative correlations with activated CD4^+^ T cells and memory B cells; DDIT4 and PDGFD were positively correlated with activated CD4^+^ T cells; MMP8 negatively correlated with memory B cells; and SPHK1 negatively correlated with activated CD4^+^ T cells (**Figures 10A–L**). These findings suggest a mechanistic link between oxidative stress and immune dysregulation in uveitis.

**Figure 8:**
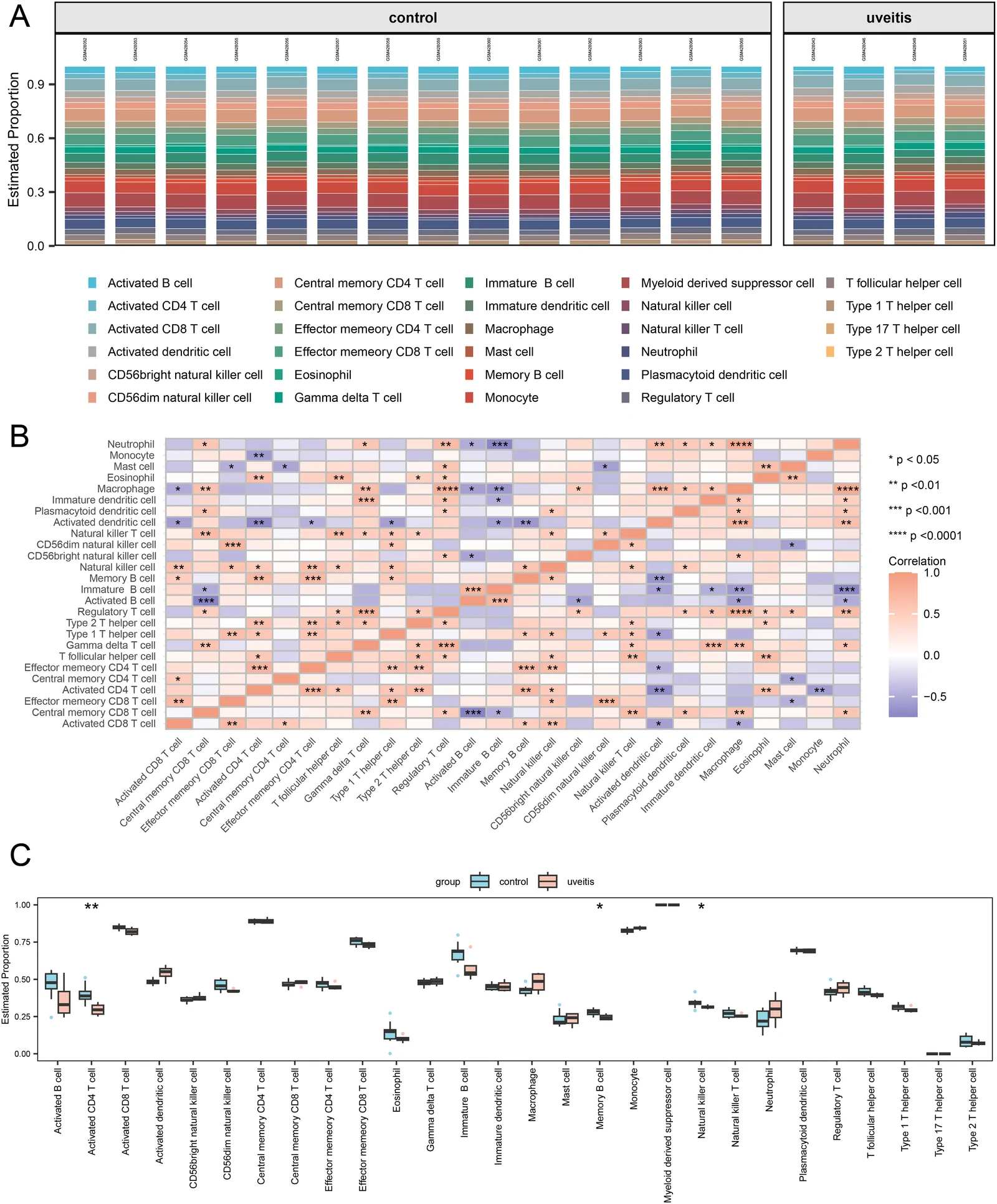
Peripheral blood immune cell abundance profiles in Behçet’s disease-associated uveitis (BU). **(A)** Stacked bar plot showing the relative proportions of 28 immune cell subsets in BU patients and healthy controls. Each bar represents an individual sample, with group annotations indicated at the top. This panel serves as a quality control visualization to confirm intragroup consistency and the absence of outlier samples. **(B)** Heatmap displaying correlation matrices among immune cell types in BU samples. Red and blue indicate positive and negative correlations, respectively. **(C)** Box plots showing immune cell subsets with significantly different relative abundances between BU and control groups (Wilcoxon rank-sum test, FDR-adjusted p < 0.05). Boxes represent the interquartile range (IQR), with the median indicated by the central line; whiskers extend to the most extreme data points within 1.5 × IQR. Statistical significance: *p < 0.05, **p < 0.01, ***p < 0.001, ****p < 0.0001.

**Figure 9:**
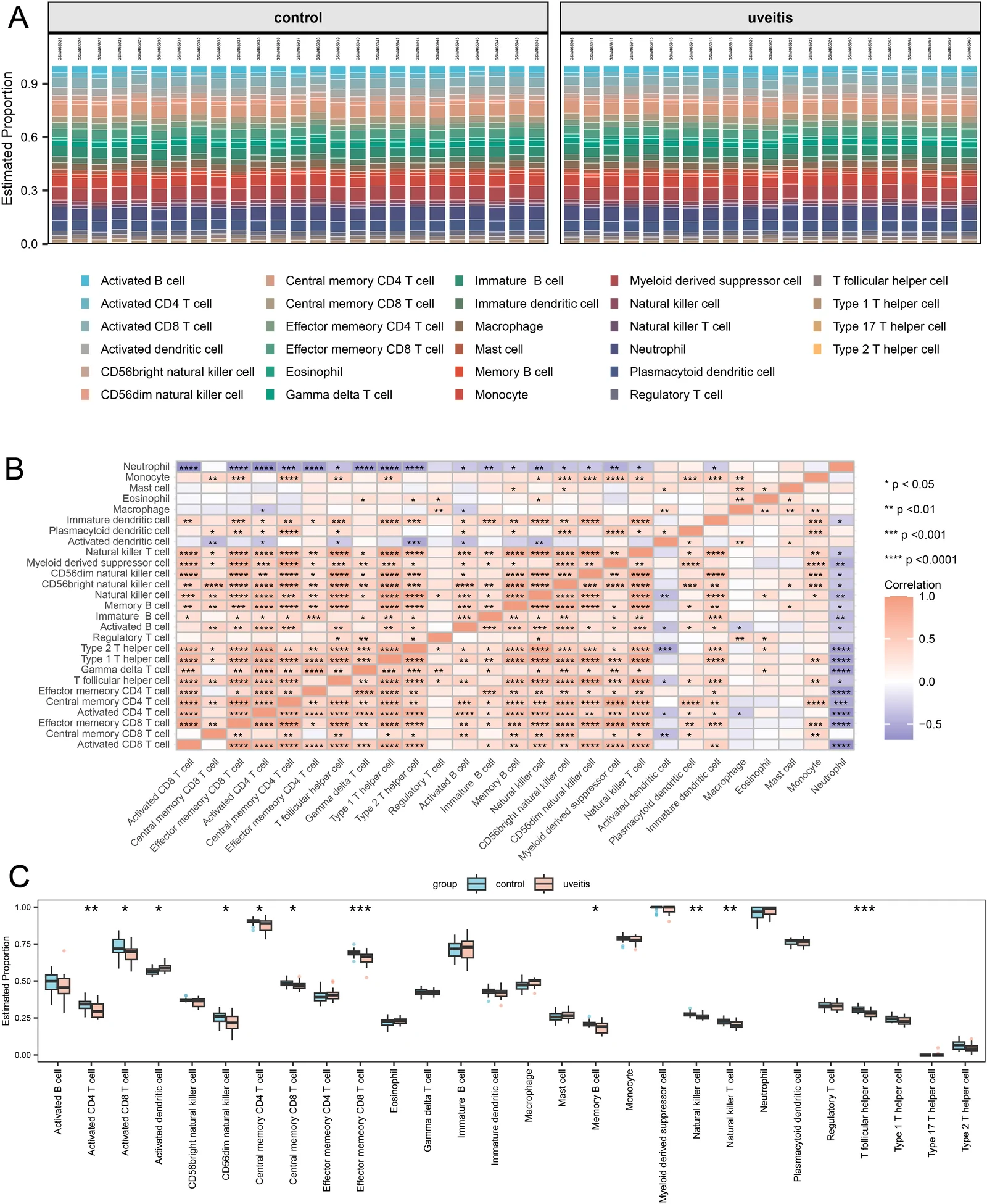
Peripheral blood immune cell abundance profiles in sarcoidosis-associated and spondyloarthropathy (SpA)-associated uveitis. **(A)** Stacked bar plot showing the relative proportions of 28 immune cell subsets in sarcoidosis/SpA uveitis patients and healthy controls. Each bar represents an individual sample, with group annotations indicated at the top. This panel serves as a quality control visualization to confirm intragroup consistency and the absence of outlier samples. **(B)** Heatmap displaying correlation matrices among immune cell types in sarcoidosis/SpA uveitis samples. Red and blue indicate positive and negative correlations, respectively. **(C)** Box plots showing immune cell subsets with significantly different relative abundances between sarcoidosis/SpA uveitis and control groups (Wilcoxon rank-sum test, FDR-adjusted p < 0.05). Boxes represent the interquartile range (IQR), with the median indicated by the central line; whiskers extend to the most extreme data points within 1.5 × IQR. Statistical significance: *p < 0.05, **p < 0.01, ***p < 0.001, ****p < 0.0001.

**Figure 10:**
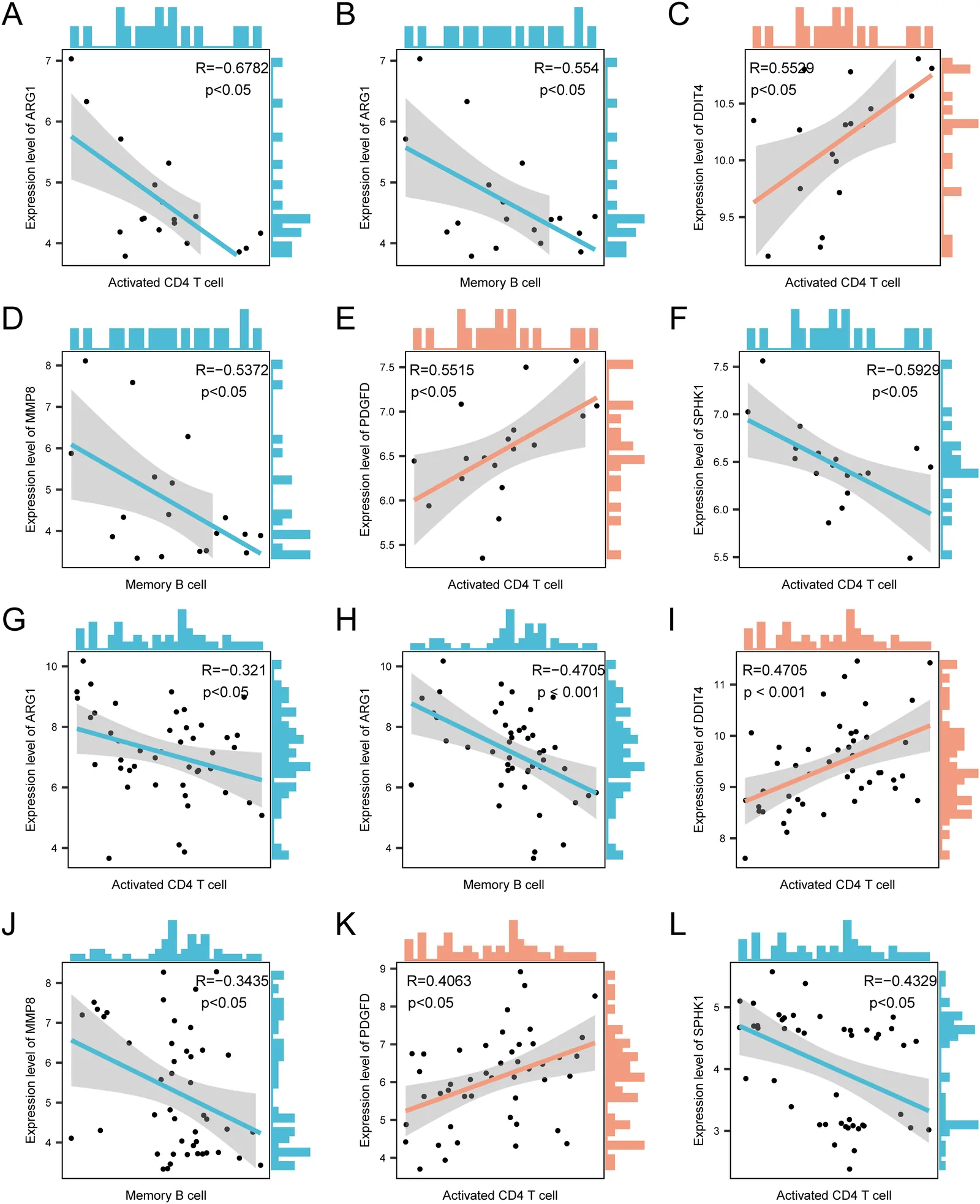
Correlation between key genes and immune cells. In BD uveitis: **(A)** ARG1 and activated CD4 T cells, **(B)** ARG1 and memory B cells, **(C)** DDIT4 and activated CD4 T cells, **(D)** MMP8 and memory B cells, **(E)** PDGFD and activated CD4 T cells, **(F)** SPHK1 and activated CD4 T cells. In Sarcoidosis & SpA uveitis: **(G)** ARG1 and activated CD4 T cells, **(H)** ARG1 and memory B cells, **(I)** DDIT4 and activated CD4 T cells, **(J)** MMP8 and memory B cells, **(K)** PDGFD and activated CD4 T cells, **(L)** SPHK1 and activated CD4 T cells.

### Regulatory network construction for OSDEGs

To gain deeper insight into the regulation of OSDEG expression, an mRNA–miRNA–lncRNA interaction network was constructed and visualized using Cytoscape. This analysis identified microRNAs targeting DDIT4 and CCR7, as well as their associated long non-coding RNAs (lncRNAs) (**Figure 11A**; **Supplementary Table S8**). Additionally, mRNA–RBP interactions were retrieved from the StarBase database, resulting in a network of 72 nodes (68 RBPs and 4 mRNAs) and 83 edges (**Figure 11B**; **Supplementary Table S9**). A transcription factor (TF)–target gene network, including ten TFs and four OSDEGs, was also constructed and visualized in Cytoscape (**Figure 11C**; **Supplementary Table S10**).

**Figure 11:**
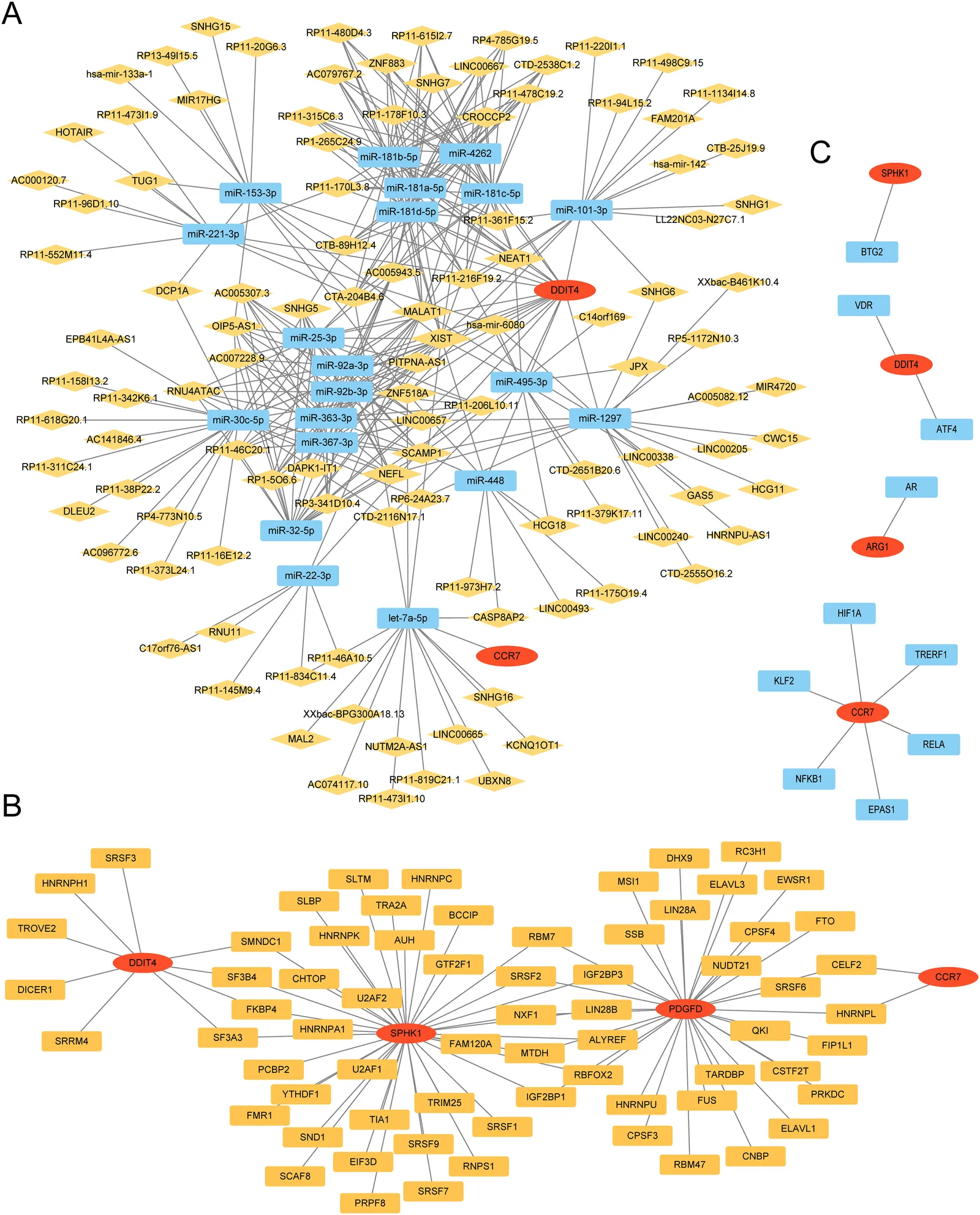
Construction of TF–mRNA, mRNA–RBP, and ceRNA networks. **(A)** lncRNA–miRNA–mRNA network of key genes: yellow represents lncRNAs, orange represents mRNAs, and sky blue represents miRNAs. **(B)** mRNA–RBP regulatory network: yellow represents RBPs, and orange represents mRNAs. **(C)** TF–mRNA interaction network of key genes: orange represents target genes, and blue represents transcription factors (TFs).

### Validation of oxidative stress-related DEGs in BU Patients

The clinical characteristics of the BU patients and healthy controls are summarized in **Supplementary Table S11**. Quantitative RT-PCR analysis revealed significant upregulation of MMP8, ARG1, and SPHK1, along with downregulation of CCR7, DDIT4, and PDGFD, in peripheral blood mononuclear cells (PBMCs) from Behçet’s uveitis (BU) patients compared to healthy controls (**Figures 12A–F**).

**Figure 12:**
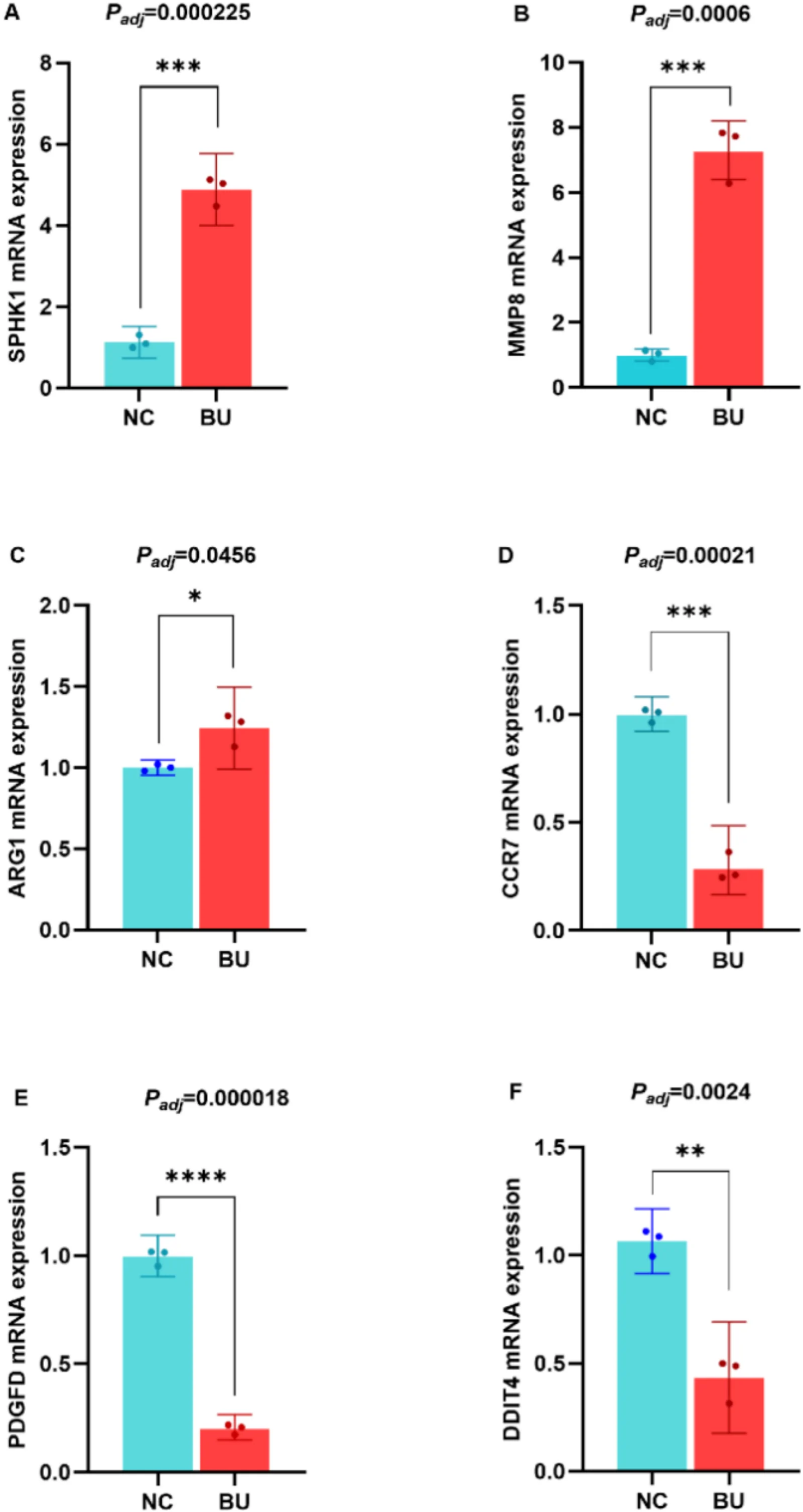
qRT-PCR Confirms oxidative stress-related DEGs expression in BU. qRT-PCR analysis revealed that SPHK1 **(A)**, MMP8 **(B)**, and ARG1 **(C)** were significantly upregulated, while CCR7 **(D)**, PDGFD **(E)**, and DDIT4 **(F)** were significantly downregulated in BD patients compared to healthy controls. *p<0.05, **p<0.01, ***p<0.001, ****p<0.0001.

### SPHK1 drives ROS-induced pyroptosis via NLRP3 inflammasome activation

Correlations between SPHK1 expression and activation of ROS-related pathways in both GSE17114 and GSE18781 datasets (**Figures 6B**, **7B**) led us to hypothesize that SPHK1 modulates inflammatory responses through ROS-dependent mechanisms. This hypothesis was supported by prior clinical observations in monocyte-derived macrophages from patients with BU and VKH syndrome, where elevated ROS levels were associated with NLRP3 inflammasome activation and increased IL-1β production, a hallmark of pyroptosis. Pyroptosis is defined by the activation of caspase-1, formation of membrane pores mediated by gasdermin D, and the release of pro-inflammatory cytokines, which collectively intensify inflammation and contribute to tissue damage. To experimentally test this hypothesis, we established an LPS-stimulated bone marrow-derived macrophage model that mimics key features of uveitis. Initial validation showed marked SPHK1 upregulation in this model (**Figure 13A**). Functional assays revealed that SPHK1 knockdown significantly reduced oxidative stress, as indicated by lower intracellular ROS (**Figure 13B**) and decreased MDA levels (**Figure 13C**). Concurrently, silencing of SPHK1 inhibited pyroptosis, leading to decreased secretion of IL-1β and IL-18 (**Figure 13D**), along with reduced activation of the NLRP3 inflammasome, ASC, cleaved caspase-1, and GSDMD (**Figure 13E**). In contrast, SPHK1 overexpression enhanced inflammasome activation and cytokine release, effects that were abolished by pre-treatment with the ROS scavenger NAC (**Figures 13F, G**), thereby confirming that SPHK1-induced pyroptosis occurs via a ROS-dependent pathway.

**Figure 13:**
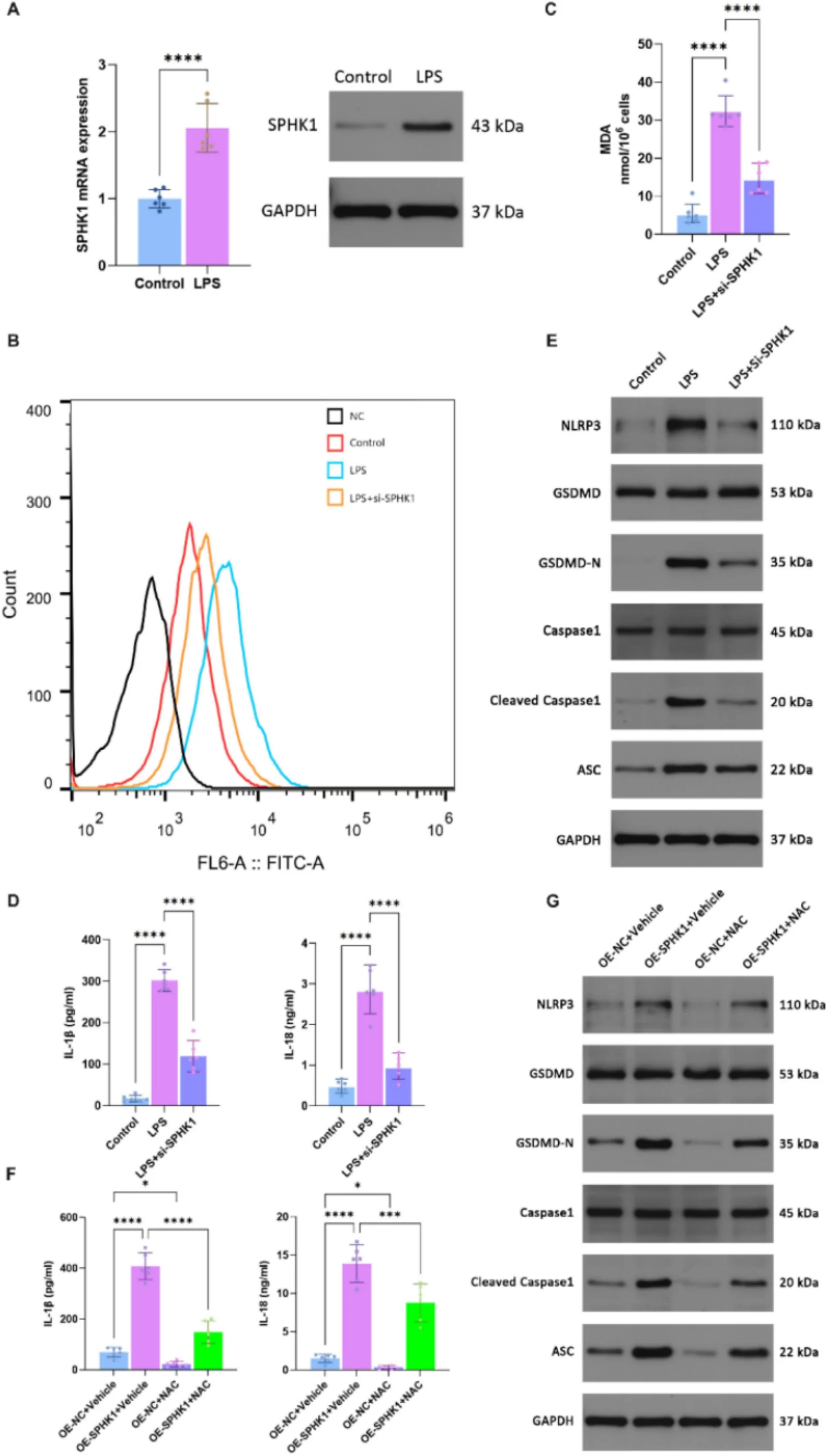
SPHK1 drives ROS-dependent NLRP3 inflammasome-mediated pyroptosis in uveitis. **(A)** SPHK1 expression was significantly upregulated in LPS-stimulated bone marrow-derived macrophages (BMDMs), as determined by qRT-PCR and Western blot analysis. **(B)** Knockdown of SPHK1 attenuated intracellular ROS production in LPS-induced BMDMs, as measured by DCFH-DA fluorescence. **(C)** SPHK1 silencing reduced malondialdehyde (MDA) content, indicating decreased lipid peroxidation. **(D)** ELISA analysis showed that SPHK1 knockdown suppressed the release of IL-1β and IL-18 in cell culture supernatants. **(E)** Western blot analysis demonstrated that SPHK1 deficiency inhibited the expression of NLRP3 and ASC, and reduced the cleavage of caspase-1 and GSDMD. **(F, G)** The enhanced production of IL-1β and IL-18 **(F)** and activation of pyroptosis-related proteins **(G)** induced by SPHK1 overexpression were reversed by pretreatment with the ROS scavenger NAC (5 mM). * p<0.05, *** p<0.001, **** p<0.0001.

### SPHK1 is a putative direct target of dexamethasone with strong binding affinity

To explore the druggability of the key hub gene SPHK1, we performed molecular docking with the widely used corticosteroid Dexamethasone. The analysis predicted a strong binding affinity (Vina Score: -7.3 kcal/mol) between SPHK1 and Dexamethasone (**Figure 14**). The binding was stabilized by diverse intermolecular forces, including hydrogen bonds (e.g., with GLY246, ARG248), hydrophobic interactions (e.g., with HIS242, LEU247, LEU324), and ionic interactions; the detailed docking parameters and interacting residues are summarized in **Supplementary Table S12**. This result suggests that SPHK1 is a putative direct target of Dexamethasone, providing a potential structural mechanism for its anti-inflammatory effects in uveitis.

**Figure 14:**
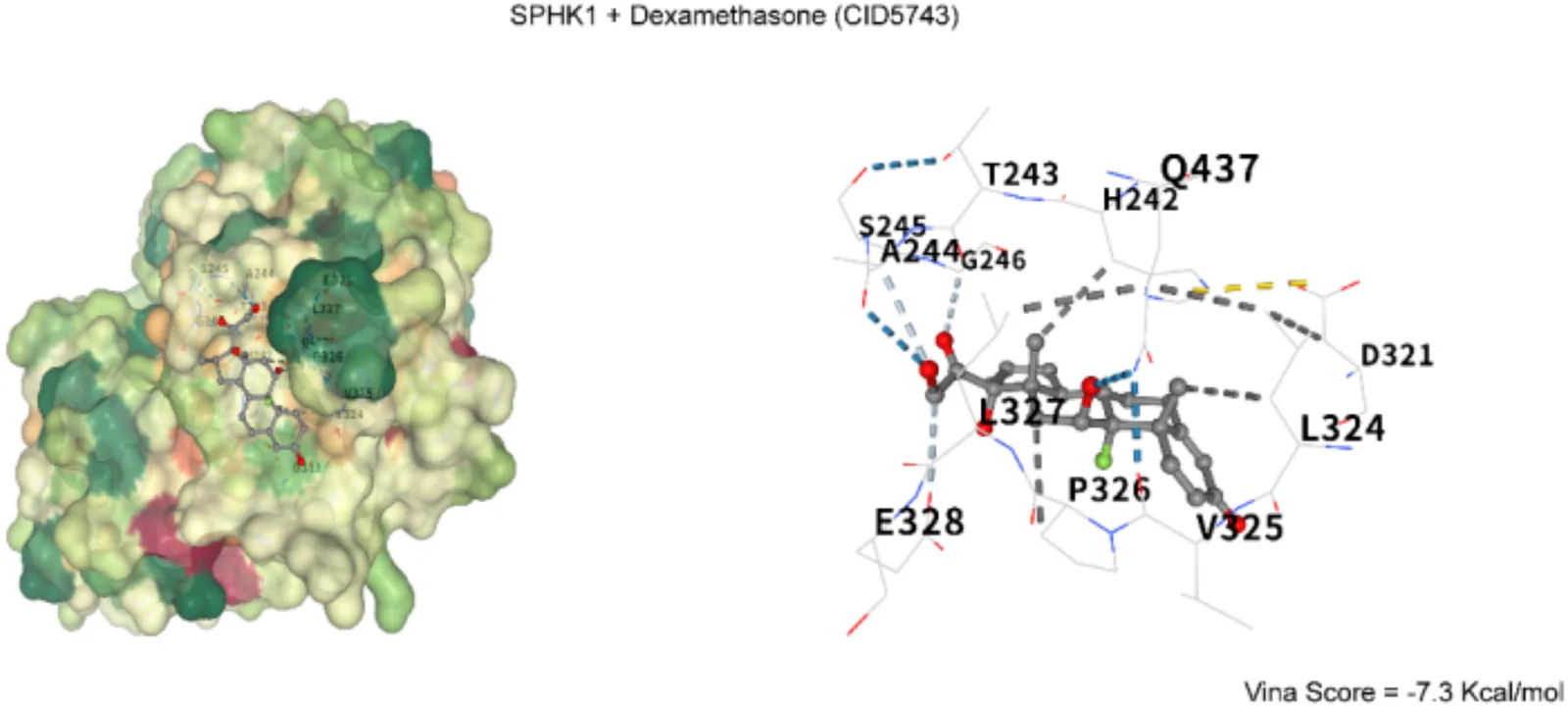
Molecular docking analysis of SPHK1 with dexamethasone. Visualization of the docking pose and interaction details between SPHK1 (PDB: 4V24) and Dexamethasone. The left panel shows the global view of Dexamethasone (shown as pink sticks) docked within the binding pocket of the SPHK1 protein surface. The protein surface is colored from green to orange to red, indicating a gradient from hydrophilic to hydrophobic regions. The right panel illustrates the two-dimensional interaction diagram. Key interactions are denoted as follows: blue dashed lines-represent hydrogen bonds, light blue dashed lines-represent weak hydrogen bonds, gray dashed lines-represent hydrophobic interactions, and yellow dashed lines-represent ionic interactions. The strong binding affinity (Vina Score = -7.3 kcal/mol) and the multitude of interactions suggest a stable binding mode.

## Discussion

Uveitis remains a significant contributor to visual impairment and blindness worldwide. However, its underlying molecular mechanisms are still incompletely understood, particularly in subtypes associated with systemic immune diseases (28). Current treatment options, such as corticosteroids and immunosuppressants, are constrained by systemic side effects, risk of disease recurrence, and a lack of curative efficacy, which highlights the need to identify novel molecular targets for improved management (29). Recent studies have established that oxidative stress is a critical factor in immune-mediated inflammatory diseases, promoting pro-inflammatory signaling cascades and amplifying tissue damage, which contributes to a chronic cycle of inflammation (30). While increased oxidative stress markers have been observed in uveitis patients, the underlying regulatory gene networks and specific pathway-level changes are still not well characterized.

The present study aimed to systematically investigate oxidative stress-related gene signatures in three non-infectious uveitis subtypes: BU, sarcoidosis-associated uveitis, and SpA-associated uveitis. We identified key immune molecular signatures for these three uveitis subtypes, specifically highlighting six oxidative stress-related differentially expressed genes (OSDEGs): MMP8, ARG1, and SPHK1 were found to be upregulated, while DDIT4, CCR7, and PDGFD were downregulated. Their diagnostic potential and regulatory network connections were also established. Importantly, beyond transcriptomic profiling, our functional validation experiments revealed that SPHK1 functions as a central pathogenic driver. SPHK1 facilitates NLRP3 inflammasome-mediated pyroptosis through mechanisms dependent on ROS. This newly characterized SPHK1–ROS–NLRP3–pyroptosis axis represents an unrecognized pathogenic cascade in non-infectious uveitis. Notably, molecular docking analysis revealed strong binding affinity between SPHK1 and dexamethasone, a first-line corticosteroid used in uveitis management, with multiple interaction sites identified at the SPHK1 active pocket. This computational evidence suggests that SPHK1 may serve as a direct target of dexamethasone, potentially explaining part of its therapeutic mechanism and positioning SPHK1 as a promising druggable target for more specific interventions. Alongside this pathway, ARG1 was shown to modulate immunometabolism, and CCR7 deficiency was linked to impaired immune homeostasis. Collectively, these oxidative stress mediators provide new mechanistic insights into the pathogenesis of non-infectious uveitis, while also offering promising diagnostic biomarkers, particularly SPHK1, ARG1, and MMP8, which displayed AUCs of 0.83 to 0.94, and therapeutic targets for more precise interventions in ocular inflammation.

SPHK1 encodes sphingosine kinase 1, a key enzyme in sphingolipid metabolism that catalyzes the conversion of sphingosine to sphingosine-1-phosphate (S1P) (21). This kinase is primarily expressed in immune-associated tissues such as the spleen, thymus, and peripheral blood, where it plays a key role in regulating inflammation, oxidative stress responses, and immune cell trafficking (22). In various autoimmune and inflammatory diseases, including rheumatoid arthritis and inflammatory bowel disease, overexpression of SPHK1 worsens disease pathology by promoting the production of pro-inflammatory cytokines (TNF-α, IL-1β, IL-6) and enhancing leukocyte infiltration via activation of the JAK2/STAT3 and NF-κB signaling pathways (23, 24). Moreover, SPHK1 promotes mitochondrial ROS production in various disease models, whereas SPHK1 inhibition attenuates oxidative injury, as demonstrated in studies of cardiac and pulmonary fibrosis (31–34). SPHK1 is also essential for regulating immune cell trafficking under inflammatory conditions. For example, in experimental autoimmune encephalomyelitis, SPHK1 inhibition effectively blocks Th17 cell migration (35). In models of endotoxin-induced uveitis, retinal SPHK1 upregulation leads to increased leukocyte adhesion and pro-inflammatory cytokine expression, which can be reversed by SPHK1 inhibition (36, 37). Our present findings extend these observations by demonstrating, for the first time, that SPHK1 triggers NLRP3 inflammasome-mediated pyroptosis in non-infectious uveitis through ROS-dependent mechanisms. Functionally, silencing SPHK1 decreased intracellular ROS accumulation and lipid peroxidation, while also inhibiting NLRP3 inflammasome assembly, caspase-1 activation, GSDMD cleavage, and the release of IL-1β and IL-18. Importantly, the use of the ROS scavenger NAC effectively reversed pyroptosis induced by SPHK1 overexpression, confirming that ROS serves as a crucial intermediary. The strong binding affinity between SPHK1 and dexamethasone predicted by our molecular docking analysis provides additional translational relevance to these findings, offering a structural rationale for targeting SPHK1 in therapeutic strategies. This docking result carries particular translational relevance for spondyloarthropathy (SpA)-associated uveitis, which is characteristically an acute anterior uveitis, often HLA-B27-associated, that typically responds rapidly to topical corticosteroid therapy and carries a favorable visual prognosis compared with posterior uveitis subtypes (38). Given that SPHK1 knockdown suppressed pyroptosis in our *in vitro* model and that dexamethasone binds SPHK1 with high affinity, corticosteroids may exert part of their anti-inflammatory effect in this subtype through SPHK1 inhibition; the anterior segment predilection of SpA-associated uveitis, where locally applied dexamethasone reaches therapeutic concentrations with minimal systemic exposure, makes this mechanism especially plausible. By contrast, Behçet’s disease- and sarcoidosis-associated uveitis frequently involve posterior segment inflammation (vitreous, retina, choroid) that is less amenable to topical therapy and often requires systemic immunomodulation (39). These considerations highlight the potential for subtype-specific treatment optimization based on anatomical disease distribution and route of steroid administration.

It is worth noting that although the present study focused on and directly measured the ROS–NLRP3–pyroptosis axis (ROS accumulation, NLRP3 assembly, caspase-1 activation, GSDMD cleavage, and IL-1β/IL-18 release), direct assessment of phosphorylated STAT3 and phosphorylated p65 NF-κB remains an important next step, given that SPHK1/S1P activates the JAK2/STAT3 pathway through S1PR1 in a feed-forward amplification loop (24) and that S1P acts as a TRAF2 cofactor to promote NF-κB activation (40). We acknowledge that molecular docking has inherent limitations, including fixed conformations, scoring-function bias, and a simplified solvent environment, and should therefore be interpreted cautiously. Nevertheless, the predicted SPHK1–dexamethasone interaction is supported by multiple independent lines of evidence: our functional data show that SPHK1 is required for pyroptosis, and experimental studies demonstrate that glucocorticoids upregulate SphK1 expression and S1P production to exert cytoprotection (41) and that SphK1 induction is required for their anti-inflammatory action (42). This corroborating evidence supports the biological plausibility of the SPHK1–dexamethasone interaction, so our therapeutic interpretation does not rest on docking alone. Further validation of this interaction by molecular dynamics simulations and direct binding assays will be pursued in future work.

Complementing the role of SPHK1 in inflammatory cell death, ARG1 emerges as a context-dependent modulator of immunometabolism (43). Our data confirms consistent ARG1 upregulation across multiple uveitis subtypes, alongside robust diagnostic value (AUC = 0.94). Although ARG1 does not directly participate in mitochondrial respiration, it depletes L-arginine, which may reduce nitric oxide synthesis and shift the redox environment toward oxidative stress. Its enrichment in TCA cycle pathways implies indirect modulation of mitochondrial metabolism through ornithine-derived metabolites (44, 45). Interestingly, while ARG1 is protective in some myeloid cell contexts, it is pathogenic in retinal glia, highlighting the need for cell-type-specific studies in the future (46, 47). MMP8 and CCR7 also contribute to non-infectious uveitis through distinct mechanisms. Elevated MMP8 derived from neutrophils in our cohorts suggests a role in tissue remodeling and complement activation, which is consistent with increased MMP8 found in the aqueous humor of uveitis-induced glaucoma (48). In contrast, CCR7 downregulation observed across uveitis subtypes is linked to impaired immune homeostasis. CCR7 expression correlates positively with oxidative phosphorylation and negatively with ROS/complement pathways, suggesting that CCR7 deficiency could disrupt Treg cell trafficking and exacerbate oxidative stress. As a G protein-coupled receptor on dendritic cells, T cells, and B cells, CCR7 directs immune cell migration and helps maintain immune homeostasis (49). In the experimental autoimmune uveitis model, reduced CCR7 expression in lymph node Treg cells was associated with diminished inflammatory control. Clinical studies similarly found that T cells from uveitis patients exhibit reduced ability to upregulate CCR7 in Treg cells (50). Altogether, our data position SPHK1 as a central orchestrator of non-infectious uveitis pathogenesis via the ROS–NLRP3–pyroptosis axis, while ARG1, MMP8, and CCR7 influence complementary aspects of immunometabolic dysfunction. The convergence of these mediators on oxidative stress pathways underscores their potential as combined therapeutic targets for managing vision-threatening inflammation.

Inflammatory immune-mediated diseases are fundamentally linked to aberrant immune responses driven by inflammatory mediators and immune cells (51). Our GO and KEGG enrichment analyses indicate that T cell activation, differentiation, and receptor signaling are key pathways involved in all three non-infectious uveitis subtypes analyzed. Immune cell abundance profiling further revealed a significant reduction in the relative abundances of activated CD4+ T cells, memory B cells, and NK cells in uveitis patients compared to healthy controls. For memory B cells and NK cells, our findings align directly with independent flow-cytometric evidence. CD27+ memory B cell is significantly reduced in BD peripheral blood and normalize after anti-TNF therapy, consistent with redistribution to inflamed tissues (52). Both CD56^Dim and CD56^Bright NK subsets are significantly depleted in BD, with disease activity and azathioprine as independent predictors (53). In sarcoidosis, peripheral CD4+ lymphopenia is a well-established correlate of progressive disease (54, 55). For activated CD4+ T cells, the evidence is more nuanced. Dagur et al. reported that CD4+CD146+ (MCAM+) Th17 cells—one subset of activated CD4+ T cells—are significantly elevated in BD and sarcoidosis peripheral blood (56), in apparent tension with the reduced relative abundance of “activated CD4+ T cells” estimated by our ssGSEA analysis. We submit that this discrepancy arises from a fundamental difference in measurement dimension, not from a genuine contradiction. Dagur et al. enumerated a specific, numerically small subset defined by a single surface adhesion molecule. By contrast, the TISIDB-derived ssGSEA gene signature captures a much broader population via a multi-gene transcriptional program integrating diverse activation states. Selective expansion of the minor CD146+ compartment can coexist with reduced relative abundance of the broader activated CD4+ T cell compartment. Moreover, ssGSEA estimates relative proportions among 28 immune cell types (compositional data); the elevated myeloid-lineage cells in uveitis samples mathematically reduce lymphoid proportions independently of absolute counts. Another important consideration is the lymphocyte redistribution hypothesis, whereby activated immune cells traffic from peripheral blood into inflamed tissues. This is supported by experimental autoimmune uveitis models, in which activated CD4+ T cells migrate across the blood-retina barrier via a CCR5-dependent mechanism (57), and provides a biologically plausible framework for reduced peripheral blood lymphoid proportions during active ocular inflammation. Interestingly, in GSE17114, three of the four BD uveitis samples were classified as immunosuppressed at blood collection; in GSE18781, by contrast, systemic immunomodulatory therapy was explicitly not permitted. Unexpectedly, the replication of the same directional immune cell abundance changes in both datasets adds to the complexity of the systemic immune microenvironment. However, our transcriptomic data alone cannot distinguish lymphocyte redistribution from compositional shifts driven by myeloid expansion or from genuine peripheral depletion. Paired peripheral blood and ocular tissue analyses in future studies will be essential to clarify these distinctions.

Despite these findings, several limitations should be acknowledged. First, the modest sample sizes of the public transcriptomic datasets (GSE17114 and GSE18781) and the absence of standardized severity grading (e.g., the SUN criteria) limit our ability to correlate DEGs expressions particularly that of SPHK1—with specific disease stages. Second, because both datasets derive from circulating immune cells, the profiles reflect systemic rather than tissue-resident changes; similarly, the qRT-PCR-validated changes in PBMCs should therefore be regarded as a peripheral blood biomarker, and whether the same changes occur in intraocular tissues (aqueous humor, vitreous, retina) remains to be determined. Moreover, restricting the analysis to confirmed uveitis patients left non-uveitis disease controls unexplored. Third, sarcoidosis- and SpA-associated uveitis derive from a single published cohort (GSE18781) in which the two entities were profiled together; following the source cohort’s definition, we analysed them as one combined group to preserve analytical robustness, so the two subtypes were not resolved independently, limiting subtype-specific interpretation. Fourth, qRT-PCR validation was restricted to BU owing to its clinical severity and sample availability, and whether the six OSDEGs carry equivalent diagnostic value in sarcoidosis- and SpA-associated uveitis awaits confirmation in additional subtype-specific cohorts. Fifth, our *in vitro* studies were limited to LPS-stimulated BMDMs—an acute, endotoxin-induced model that does not recapitulate the chronic, recurrent, T-cell-mediated features of autoimmune uveitis—and to a single macrophage lineage; given that SPHK1/S1P signalling also regulates CD4^+^ T-cell and dendritic cell function, confirmation of the SPHK1–ROS–NLRP3–pyroptosis axis in T-cell-mediated experimental autoimmune uveitis models, in additional cell types, and in human ocular tissue is warranted. Notwithstanding these limitations, our study provides robust evidence identifying SPHK1 as a novel driver of pyroptosis in non-infectious uveitis through ROS-dependent NLRP3 inflammasome activation, and future studies with larger subtype-specific cohorts will be needed to translate these findings into precision diagnosis and targeted therapy.

## Conclusion

In summary, this integrated study identified six oxidative stress-related mediators, including SPHK1, MMP8, ARG1, CCR7, PDGFD, and DDIT4, that possess significant diagnostic value in autoimmune uveitis. Importantly, our functional experiments demonstrated that SPHK1 is a central driver of disease, as it promotes NLRP3 inflammasome-mediated pyroptosis through a mechanism dependent on reactive oxygen species. This discovery establishes a novel pathogenic axis implicated in the onset and progression of uveitis. Additionally, our results indicate that ARG1 plays a role in regulating immunometabolism, while CCR7 deficiency contributes to impaired immune homeostasis. Both mechanisms are critical in sustaining ocular inflammation. By elucidating these interconnected molecular pathways, our research provides new insights into the complex mechanisms underlying uveitis pathogenesis. These findings provide a solid foundation for future research focused on developing targeted therapeutic strategies that address oxidative stress–driven immune dysfunction and ultimately enhance clinical outcomes for patients with vision-threatening autoimmune uveitis.

## Data Availability

The datasets presented in this study can be found in online repositories. The names of the repository/repositories and accession number(s) can be found below: Gene Expression Omnibus (GEO), accession numbers GSE17114 and GSE18781 (https://www.ncbi.nlm.nih.gov/geo/). The experimental data generated in this study are included in the article/Supplementary Material. Further inquiries can be directed to the corresponding author.

## Supplementary material

Supplementary Table 1 Oxidative stress (OS)-related genes.

Supplementary Table 2 Differentially expressed genes in BD uveitis samples (n = 660 DEGs).

Supplementary Table 3 Differentially expressed genes identified in sarcoidosis & SpA uveitis versus control samples (n = 1161 DEGs).

Supplementary Table 4 Protein–protein interaction (PPI) network of key genes constructed via GeneMANIA: 6 key genes and 20 co-expressed genes.

Supplementary Table 5 GO enrichment analysis of the 26 co-expressed genes identified in the PPI network.

Supplementary Table 6 Associations between peripheral blood immune cell abundances and BD uveitis versus control samples.

Supplementary Table 7 Associations between peripheral blood immune cell abundances and sarcoidosis & SpA uveitis versus control samples.

Supplementary Table 8 Construction of mRNA–miRNA–lncRNA regulatory network based on DDIT4 and CCR7 using cytoscape.

Supplementary Table 9 RBP–mRNA interaction network of key genes constructed via StarBase: 72 Nodes (68 RBPs, 4 mRNAs) and 83 edges.

Supplementary Table 10 Transcription factor–mRNA network of key genes constructed using hTFtarget and visualized with cytoscape.

Supplementary Table 11 Clinical characteristics of the BU validation cohort.

Supplementary Table 12 Molecular docking results demonstrating strong binding of Dexamethasone to SPHK1.
