# Supplementary material for: SPHK1 drives pyroptosis via ROS-dependent activation of the NLRP3 inflammasome in non-infectious uveitis
Source: Front Immunol. 2026 Sep 29;17:1764448. doi: 10.3389/fimmu.2026.1764448 (PMC13657954; doi:10.3389/fimmu.2026.1764448)
Supplement: Supplementary file 1 [file DataSheet1.zip › Tables/table S11Clinical characteristics of the BU validation cohort.pdf]

Table S11: Clinical data of participants recruited in the study.

**Characteristics**	**Behçet's uveitis (BU) (n=3)**	**Healthy control (HC) (n=3)**
Age (years), median (range)	35 (21–46)	36 (28–51)
Gender (M/F)	2/1	2/1
**Ocular manifestations**		
Anterior chamber flare		
(Grade 1+/Grade 2+)	1/2	0 (0)
Anterior chamber cells		
(Grade 1+/Grade 2+/Grade 3+)	1/1/1	0 (0)
Posterior synechiae, n (%)	2 (66.7)	0 (0)
Hypopyon, n (%)	2 (66.7)	0 (0)
Keratic precipitates (KPs), n (%)	3 (100)	0 (0)
Vitreous haze, n (%)	2 (66.7)	0 (0)
Retinal vasculitis, n (%)		
(Grade 1+/Grade 2+/Grade 3+)	1/1/1	0 (0)
Macular edema, n (%)	1 (33.3)	0 (0)
**Systemic manifestations**		
Oral ulcers, n (%)	3 (100)	0 (0)
Genital ulcers, n (%)	2 (66.7)	0 (0)
Cutaneous lesions, n (%)	2 (66.7)	0 (0)
Arthritis, n (%)	2 (66.7)	0 (0)

M: male; F: female.
